# Polymeric Guide Conduits for Peripheral Nerve Tissue Engineering

**DOI:** 10.3389/fbioe.2020.582646

**Published:** 2020-09-25

**Authors:** Huiquan Jiang, Yun Qian, Cunyi Fan, Yuanming Ouyang

**Affiliations:** ^1^College of Fisheries and Life Science, Shanghai Ocean University, Shanghai, China; ^2^Department of Orthopedics, Shanghai Jiao Tong University Affiliated Sixth People’s Hospital, Shanghai, China; ^3^Shanghai Sixth People’s Hospital East Affiliated to Shanghai University of Medicine & Health Sciences, Shanghai, China

**Keywords:** peripheral nerve regeneration, tissue engineering, nerve guide conduit, scaffold material, polymer

## Abstract

Peripheral nerve injuries (PNIs) are usually caused by trauma, immune diseases, and genetic factors. Peripheral nerve injury (PNI) may lead to limb numbness, muscle atrophy, and loss of neurological function. Although an abundance of theories have been proposed, very few treatments can effectively lead to complete recovery of neurological function. Autologous nerve transplantation is currently the gold standard. Nevertheless, only 50% of all patients were successfully cured using this method. In addition, it causes inevitable damage to the donor site, and available donor sites in humans are very limited. Tissue engineering has become a research hotspot aimed at achieving a better therapeutic effect from peripheral nerve regeneration. Nerve guide conduits (NGCs) show great potential in the treatment of PNI. An increasing number of scaffold materials, including natural and synthetic polymers, have been applied to fabricate NGCs for peripheral nerve regeneration. This review focuses on recent nerve guide conduit (NGC) composite scaffold materials that are applied for nerve tissue engineering. Furthermore, the development tendency of NGCs and future areas of interest are comprehensively discussed.

## Introduction

Peripheral nerve injuries (PNIs) are common clinical cases. These are usually caused by trauma, immune diseases (e.g., lupus erythematosus), and genetic factors (e.g., mutation in LAMA2). Damage to the peripheral nerves may cause nerve defects, breakage of internal blood vessels, and interruption of the interaction between neurons and tissues (Domínguez et al., 2013). Peripheral nerve defects, which are common in PNIs, can lead to limb numbness and muscle atrophy, and to the loss of neurological function (Lim et al., 2017).

**FIGURE 1:**
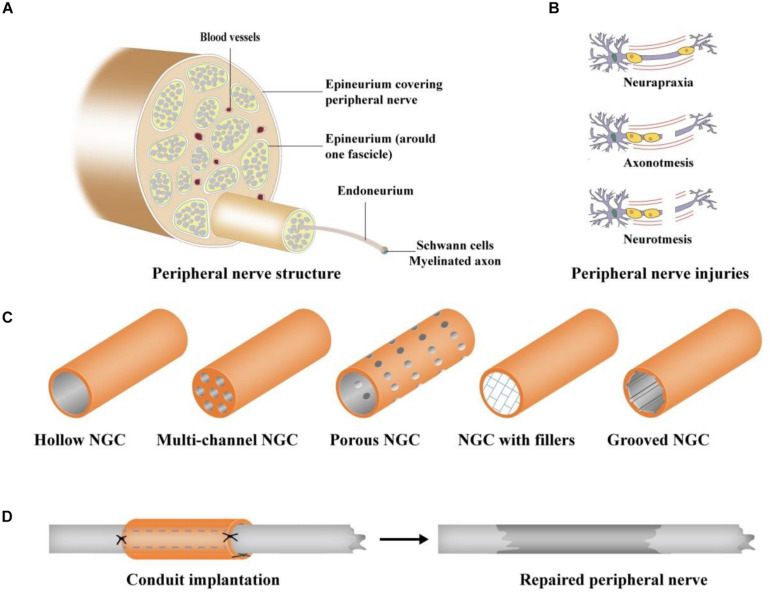
Schematic illustration of PNI and NGCs (Vijayavenkataraman, 2020). **(A)** Peripheral nerve structure, **(B)** different types of PNI: Neurapraxia, Axonotmesis, and Neurotmesis; **(C)** different designs of NGCs: hollow, multi-channel, porous, grooved NGC, and NGC with fillers, and **(D)** surgical procedure of NGC grafting.

Neurorrhaphy is usually applied to suture the nerve gap of the peripheral nerve injury (PNI) when the gap of the injured peripheral nerve is less than 5 mm (Li et al., 2013). When the nerve gap is greater than 5 mm, the direct end-to-end suture is not suitable for nerve repair (Li et al., 2013). Autologous nerve transplantation as the gold standard has achieved the best effect on the treatment of PNI; however, only 50% of cases have been successful (Manoukian et al., 2019a). In addition, autologous nerve transplantation may cause inevitable damage to the donor site and neuroma formation (Oprych et al., 2016). Furthermore, available donor sites in humans are very limited (Bozkurt et al., 2015). To achieve a better therapeutic effect on peripheral nerve regeneration, tissue-engineered grafting has been increasingly applied for the treatment of PNI as a potential treatment method (Huang et al., 2017). Tissue engineering, which is dedicated to artificial tissues and organs, arises at the historic moment. With more than 30 years of development, tissue engineering has played an extremely significant role in tissue or organ repair (Almansoori et al., 2020). Numerous studies (Oprych et al., 2016; Lackington et al., 2017; Vijayavenkataraman, 2020) have demonstrated that nerve guide conduits (NGCs) (Figure 1), as a kind of tissue-engineered implant, have enormous potential to bridge injury sites and provide a physical template for peripheral nerve regeneration. Various scaffold materials have been applied to prepare NGCs that mimic the natural microenvironment of peripheral nerves to guide neural outgrowth and extension (Song et al., 2018; Zhu et al., 2020).

Polymeric scaffold materials, as cell and active molecular carriers, are particularly important in tissue engineering (Sanjairaj et al., 2017). Polymeric scaffold materials mainly include natural and synthetic polymers (Titorencu et al., 2016). NGCs made of natural polymers usually have excellent biocompatibility and can promote the adhesion and growth of cells. Synthetic polymers have good mechanical properties for fabrication into three-dimensional (3D) scaffolds (Sanjairaj, 2016). A suitable scaffold material should be beneficial for cell growth, axonal regeneration, and for rebalancing of the microenvironment with its superior mechanical properties. Additionally, it should play a role in inducing cell-specific differentiation. Thus, the biocompatibility, mechanical properties, and biodegradability of scaffold materials are primary indicators for judging the quality of different scaffold materials (Sanjairaj, 2016; Zhang et al., 2018; Zhang S. et al., 2019). Researchers have mixed natural polymers with synthetic polymers to overcome the shortcomings of pure polymer scaffold materials. Composite NGCs are new scaffolds that are composed of two or more different kinds of biomaterials according to specific proportions and methods (Vijayavenkataraman, 2020).

This review discusses recent polymeric scaffold materials (Table 1) that are applied in the fabrication of NGCs and their achievements in peripheral nerve regeneration. Moreover, we infer to prospects of these biomaterials and future development tendencies of NGCs.

**TABLE 1:** Recent polymer scaffolds for peripheral nerve tissue engineering.

**Scaffold**	**Type**	**Biocompatibility**	**Biodegradability**	**Mechanical properties**	**Electrical conductivity**	**Preparation method**	**References**
Collagen	Natural	++	+++	+		4S-StarPEG	Foidl et al., 2018
Chitosan	Natural	++	+++	+		Freeze drying	Shrestha et al., 2018
Alg	Natural	++	++	+		3D printing	Wu et al., 2019
SF	Natural	++	++	++		Freeze drying	Chien-Yu et al., 2016
PCL	Synthetic	+	+	+++		Electrospinning	Mobini et al., 2017
PLLA	Synthetic	+	+	++		Electrospinning	Lasprilla et al., 2011
PLGA	Synthetic	+	+	++		Electrospinning	Gentile et al., 2014
PPy	Synthetic	+	+	+	+	Electrochemical polymerization	Guo et al., 2013
PVDF	Synthetic	+	+	++	++	Immersion precipitation/N-TIPS	Abzan et al., 2019
GelMA	Synthetic	++	++	++		DLP printing	Lei et al., 2018
Collagen/HA-Tyr hydrogel	Natural composite	++	+++	++		3D printing	Frayssinet et al., 2020
SF/Alg	Natural composite	++	+++	+		Freeze drying	Jiao et al., 2017
Cellulose/SPI	Natural composite	++	+++	+		Chemical precipitation	Gan et al., 2016
Chitosan/Collagen	Natural composite	++	+++	++		Lyophilization and phase separation	Si et al., 2019
Collagen/PCL	Natural-synthetic composite	++	++	++		Freeze drying	Yu et al., 2011
BC/PCL	Natural-synthetic composite	++	++	+++		Electrospinning	Altun et al., 2019
GO/PCL	Synthetic composite	++	++	+++	+	Integration molding	Qian et al., 2018
rGO/ApF/PLCL	Natural-synthetic composite	++	++	++	++	Electrospinning	Wang et al., 2018
cGO/PPy/PLLA	Synthetic composite	++	+	++	++	Electrochemical deposition	Chen et al., 2019
PVDF/PCL	Synthetic composite	++	+	+++	++	Cast/annealing-solvent displacement	Cheng et al., 2020
PVDF/rGO	Synthetic composite	++	+	++	++	*In situ* thermal reduction	Abzan et al., 2019

*+, poor; ++, good; +++, excellent.*

## Natural Polymer-Based Conduits

Natural polymers have a wide application in tissue engineering because of their superior biodegradability and biocompatibility (Jiang et al., 2019). Collagen is a common structure that is highly concentrated in bone, skin, tendon, and vascular structures, and collagen type I is highly conserved (Friess, 1998) in different species. Collagen is an ideal polymer that may be used to fabricate NGCs because of its low immune response and high absorbability in the body (Liu et al., 2019), and has been commonly utilized as a carrier for drug delivery (Manoukian et al., 2019b; Qiu et al., 2020). Therefore, collagen is often used to fabricate NGCs for peripheral nerve regeneration. Bozkurt et al. (2008) used the freeze-drying method to prepare highly oriented 3D porcine collagen scaffolds and found excellent regeneration of Schwann cells (SCs) *in vitro*. Functional NGCs that support nerve cells and stem cells and NGCs loaded with drugs and nerve growth factors (NGF) are areas of interest for the future (Dan et al., 2018; Zhang et al., 2020). Collagen type-I matrix scaffolds loaded with SCs were fabricated by Georgiou et al. (2013), and the results showed that these scaffolds could promote the axonal regeneration in 5 mm defect rat sciatic nerve injury models. Foidl et al. (2018) prepared collagen scaffolds using the 4S-Starpolyethyleneglycole (4S-StarPEG) method as a cross-linker and found that the collagen loaded with NGF could release NGF slowly during the process of time. In conclusion, collagen, which may be loaded with SCs and NGF, shows great potential for peripheral nerve regeneration. Furthermore, the 3D printed hydrogel scaffolds showed excellent mechanical properties. Frayssinet et al. (2020) prepared collagen/Tyramine HA derivative (HA-Tyr) hydrogel scaffolds and found that hydrogels could maintain the vitality of cells.

The reactivity of the amino group in chitosan is stronger than that in chitin, which results in the polysaccharide having an excellent biological function and renders it chemically modifiable. Therefore, chitosan is considered to be a functional biomaterial with greater application potential than cellulose (Li et al., 2018). Owing to its ability to facilitate the attachment and proliferation of Schwann cells, chitosan has great potential for peripheral nerve regeneration (Wach et al., 2020). Owing to the lack of cues for inducing cell migration and differentiation on the chitosan scaffold surface, pure chitosan scaffolds fail to meet expectations (Shrestha et al., 2018). Si et al. (2019) prepared chitosan/collagen composite NGCs through lyophilization and a phase separation composite method. Their study demonstrated that the addition of chitosan resulted in an increase in the mechanical properties of the scaffolds.

Alginate (Alg) is a common natural polymer, which is an anionic and hydrophilic polysaccharide that has adjustable biodegradability and drug release properties (Wu et al., 2010; Zuo et al., 2015). Alg can crosslink with divalent metal ions, especially Ca^2+^, to form hydrogels in a solution (Roca et al., 2020). Many studies have demonstrated that natural polymer-based hydrogels can promote the growth of nerve cells and axonal extension for peripheral nerve regeneration (Wu et al., 2019). Hydrogels can provide the nerve with a suitable 3D microenvironment owing to the similar characteristics of nerve tissue *in vivo*. The hydrogel expands to a soft biomaterial by absorbing fluid, which results in the soft and elastic hydrogel minimizing irritability to the neighboring tissues for nerve regeneration (Samadian et al., 2020). The hydrogel could be fabricated as scaffolds of excellent mechanical properties for peripheral nerve regeneration via the 3D-printing method.

Silk fibroin (SF) is a non-toxic and non-immunogenic structural protein (Tang et al., 2012; Kundu et al., 2013). SF has superior mechanical properties compared to other natural polymers (Chien-Yu et al., 2016). Jiao et al. (2017) fabricated SF/Alg composite scaffolds loaded with NGF, which significantly improved the motor function of rats, via a freeze-drying method. Gan et al. (2016) developed another nerve guide conduit (NGC) made of cellulose and soy protein isolate (SPI) through a chemical precipitation method that successfully bridged and repaired the nerve defect in 10 mm defect rat sciatic nerve injury models. The cellulose/SPI scaffolds showed a great effect on peripheral nerve regeneration.

## Synthetic Polymer-Based Conduits

Synthetic polymers are widely used to fabricate NGCs for peripheral nerve regeneration because they have better mechanical properties to be easily fabricated into 3D structures compared to natural polymers. The recovery period of PNI is usually approximately 3–6 months (Rhatomy et al., 2019); thus, a second surgery could be avoided using biodegradable synthetic polymers. Poly (ε-caprolactone) (PCL), an FDA-approved material, is one of the most common synthetic polymers used for the fabrication of NGCs. PCL has excellent processability and compatibility (Mobini et al., 2017). The NGCs made of PCL have excellent mechanical properties to offer topographical cues to axons for peripheral nerve regeneration; however, poor biodegradability is a major shortcoming (Rhatomy et al., 2019) for the wide application of PCL. Sun et al. (2006) transplanted PCL (Mw ¼ 66,000) scaffolds into rats and found that scaffolds remained structurally intact for 24 months. Poly(acrylic acid) (PAA), an FDA-approved anionic polymer, has been widely used for tissue engineering (Jaiswal and Koul, 2013; Meng et al., 2015). The cortical gel layer has the function of cation-exchange, which could affect nerve excitation and conduction (Noor Fitrah et al., 2019). However, PAA has poor mechanical properties and is difficult to fabricate into scaffolds (Qiao et al., 2018).

Although poly(L-lactic acid) (PLLA) has a higher degradation rate than PCL, the NGCs fabricated using PCL are harmful to the body because of their high crystallinity (Frydrych et al., 2015). Poly(lactic-co-glycolic acid) (PLGA) is a synthetic polymer that combines PLLA and polyglycolic acid (PGA), and the degradation of PLGA can be increased by decreasing the ratio of PLLA (Gentile et al., 2014). PLGA (PAA: PLLA 50:50) scaffolds have been widely used for fabricating NGCs, and these NGCs have been demonstrated to promote axonal regeneration (Lackington et al., 2019). The degradation byproducts of PLGA are lactic and glycolic acid that have high acidity, and metabolization is difficult *in vivo* (Kühne et al., 2018). However, PLGA, as a widely studied material, has been used in many commercial conduits without causing metabolic problems due to the degradation byproducts.

Numerous experiments have demonstrated that the proliferation and growth of nerve cells and axonal extension could be improved through appropriate electrical stimulation (ES) (Li et al., 2008). Therefore, the electrical conductivity of scaffold materials is equally important. Conducting polymers () as biocompatible biomaterials possess cell adhesion, excellent mechanical properties, and adjustable surface hydrophilicity (Jing et al., 2018; Sadeghi et al., 2018). CPs can directly condition the state and migration of cells, transmit electrical signals, and provoke ES (Wu et al., 2016). Common CPs include polypyrrole (PPy), polythiophene (PT), and poly(p-phenylene-vinylene) (PPV). PPy is one of the most important CPs that can be fabricated into NGCs for peripheral nerve regeneration. Schmidt et al. (1997) prepared PPy scaffolds that can promote the extension of neurites and the differentiation of nerve cells. Although PPy possesses semiconductivity and readily modifiable surfaces, the short-time conductivity of PPy did not adequately support *in vivo* ES, and poor biodegradability would inevitably cause a second surgical procedure (Marakova et al., 2019).

Piezoelectric polymers are a type of special material that can produce variable electrical charges on surface under mechanical distortion without energy sources or electrodes (Ribeiro et al., 2014). Therefore, mechanical properties can be transformed into electrical properties without external electrical devices, electrodes, etc. Yang et al. (2005) evaluated a large number of polymers and ceramics to provide peripheral nerve regeneration with piezoelectric nerve conduits, and polyvinylidene fluoride (PVDF) was found as a promising polymer. PVDF is a strong piezoelectric material; thus, it has often been reported to fabricate NGCs in service documents. PVDF possesses superior properties, including great chemical resistance, piezoelectric behavior, and thermal stability (Cui et al., 2013; Tsonos et al., 2015). PVDF has been demonstrated to improve axonal regeneration for central nerve injuries and PNI (Lee et al., 2016). Guo et al. (2012) reported that PVDF nanoscaffolds promoted the activity and function of fibroblasts. Lins et al. (2016) found that PVDF could be used for NGCs to simulate the native extracellular matrix and found the growth and differentiation of monkey neural stem cells into neuronal and glial cells. Lee et al. (2011) prepared electrospun fibrous scaffolds using PVDF–trifluoroethylene (PVDF–TrFE). The results showed that the dorsal root ganglion neurons attached to scaffolds and the neurites extended to them in repeated experiments. Non-solvent induced phase separation (NIPS) and thermally induced phase separation (TIPS) are both efficient and controllable ways to fabricate NGCs with an interconnected porous network for peripheral nerve regeneration (Yousefi et al., 2014). Theoretically, the TIPS method removes thermal energy from the dope solution, whereas the NIPS method promotes solvent and non-solvent interactions (Jung et al., 2016). Abzan et al. (2018) fabricated porous PVDF scaffolds using the N-TIPS method, and the PVDF scaffolds with a higher amount of the β phase promoted better the cell attachment and proliferation of PC12 cells. In conclusion, PVDF is a suitable scaffold material for peripheral nerve regeneration. However, a second surgical procedure may be inevitable because of the poor biodegradation of PVDF (Abzan et al., 2019).

## Composite Polymer Scaffolds

To overcome the above shortcomings, researchers have mixed natural polymers with synthetic polymers and found that blends possess both good mechanical properties and cellular affinity. Researchers blended collagen and PCL to fabricate compound scaffolds, SCs were used for *in vitro* studies, and 8 mm long gap sciatic nerve injuries were used for *in vivo* studies. These studies demonstrated that collagen/PCL scaffolds had a better effect than pure PCL scaffolds on nerve regeneration (Yu et al., 2011). Electrospun matrices, which interact with cells, are ideal for cell adhesion and proliferation (Cui et al., 2019; Zhang J. et al., 2019). Altun et al. (2019) prepared BC/PCL blend nanofibrous scaffolds via the electrospinning method and the aligned BC/PCL nanoscaffolds significantly improved neurite outgrowth.

To enhance the electrical conductivity of NGCs, many electrically conductive carbon-based nanostructures have been mixed into varieties of scaffolds. Graphene (Gr) and its derivatives possess superior mechanical properties and electrical conductivity and have been demonstrated to be the most suitable biomaterials for use in peripheral nerve regeneration (Hua et al., 2009; Issa et al., 2016). Kurapati et al. (2018) found that Gr and its derivatives could be degraded into smaller nanosheets by activated neutrophils; hence, demonstrating their excellent biodegradability. Therefore, Gr could be combined with other polymers to enhance their mechanical and electrical properties. Gr and reduced graphene oxide (rGO) are electrically conductive materials. Wang et al. (2018) coated rGO on the Antheraea pernyi silk fibroin (ApF)/[Poly(L-lactic acid-co-caprolactone)] (PLCL) nanoscaffolds fabricated through the *in situ* redox reaction of rGO. The rGO/ApF/PLCL scaffolds remarkably promoted the migration and proliferation of SCs and induced the differentiation of PC12 cells.

Although graphene oxide (GO) is substantially a non-conductive material, it possesses higher compatibility with polymeric biomaterials and is easier to handle with superior dispersion in solutions than Gr. (Ning et al., 2011; Nezakati et al., 2014). Qian et al. (2018) invented a GO/PCL nanoscaffold using the integration-molding method and evaluated the regenerative effects of this scaffold in a 15 mm defect rat sciatic nerve injury model for the first time. The results showed prominent angiogenesis and satisfactory axon and myelin repair. The carboxyl groups of carboxylic graphene oxide (cGO) GO can be easily handled and have super dispersion in solutions when compared with graphene, and carbon nanotubes possess better surface hydrophilicity than GO; hence, Chen et al. (2019) fabricated cGO/PPy/PLLA composite scaffolds through the electrochemical deposition method. However, the application for peripheral nerve regeneration is limited owing to the agglomeration and weak compatibility of carbon nanotubes (Chen et al., 2019).

PVDF as a piezoelectric material has been widely used in tissue engineering, and it has high piezoelectric properties and great biocompatibility (Wang et al., 2017; Surmenev et al., 2019). The self-generating ability of PVDF provides researchers with new ideas for peripheral nerve regeneration. To achieve scaffolds with better biocompatibility, biodegradation, and mechanical properties, researchers designed a composite NGC that blended PCL with PVDF. Cheng et al. (2020) used the cast/annealing-solvent displacement method to fabricate porous PVDF/PCL composite scaffolds, and these NGCs successfully bridged the 15 mm defect in rat sciatic nerve injury. Abzan et al. (2019) fabricated PVDF/GO composite scaffolds for peripheral nerve regeneration through the NIPS method, and the incorporation of 5 wt% GO remarkably enhanced the piezoelectricity of the scaffolds. To improve the properties of NGCs, researchers have mixed many carbon-based nanostructures into PVDF membranes to fabricate composite scaffolds. Pei et al. (2015) prepared PVDF/RGO nanocomposite scaffolds using the *in situ* thermal reduction technology and found that RGO enhanced the formation of the β-phase and hydrophilicity of PVDF scaffolds.

## Discussion

Tissue-engineered grafting is a potential treatment method has been increasingly applied to the treatment of PNI to achieve a better therapeutic effect on peripheral nerve regeneration. Several scaffold materials have been used to fabricate NGCs for peripheral nerve regeneration. Natural polymers possess superior biocompatibility and biodegradability; however, their poor mechanical properties make them difficult to fabricate into 3D scaffolds. Synthetic polymers with superior mechanical properties have been widely used to fabricate 3D NGCs for peripheral nerve regeneration. Nevertheless, their poor biocompatibility limits the application of pure synthetic scaffolds for peripheral nerve regeneration. Composite scaffold materials have enormous potential to enhance the properties of traditional biomaterials. Natural-synthetic composite scaffolds possess both great mechanical properties and cellular affinity, and a large number of studies have shown that composite scaffolds have great development prospects in tissue engineering. Numerous studies have demonstrated that NGCs have enormous potential for peripheral nerve regeneration, although the achievement of NGCs has not yet surpassed that of autologous nerve transplantation. This is the reason why autologous nerve transplantation continues to be the gold standard of PNI treatment.

Although there have been numerous studies on the mechanisms of PNI and rat sciatic nerve injury models, there is no alternative treatment method for peripheral nerve long segment defects. Thus, it is urgent to develop an ideal NGC approved by the FDA that possesses excellent biocompatibility, biodegradability, mechanical properties, and electrical conductivity to be applied to the human body. As mentioned above, natural polymers promote axonal regeneration owing to the delivery of nerve cells, NGF, and drugs. Synthetic polymers have provided NGCs with superior mechanical properties or electrical conductivity. Thus, polymeric materials have great potential for peripheral nerve regeneration. The piezoelectric polymer is a type of special material that could produce variable electrical charges on the surface under mechanical distortion without energy sources or electrodes. However, the poor biodegradability of piezoelectric polymers require a second surgical procedure. Additionally, the development of piezoelectric polymers with improved biodegradability and biocompatibility is a current hotspot for peripheral nerve regeneration materials research. 3D printing is a fabrication technology that precisely dispenses cell-laden biomaterials for the construction of complex 3D functional tissues or artificial organs. DLP printing, as a type of 3D printing, makes the application of pure GelMA NGCs possible, which supports the migration and proliferation of PC12 cells. This may mean that not only could DLP printing be applied for peripheral nerve regeneration, but additional 3D printing methods could also increase the performance of pure polymer scaffolds. The exploitation of more efficient printing methods compared to 3D-based printing methods has become a recent point of interest.

Functional scaffold materials that could support nerve cells and stem cells, NGCs loaded with drugs and nerve growth factors, and 4D printing methods are areas of interest for the future. Therefore, functionalized NGCs comprise the most anticipated breakthrough materials for the treatment of PNI.

## Author Contributions

YQ and HJ conceptualized the study. HJ, YQ, CF, and YO reviewed the literature and designed the figures and tables. HJ drafted the manuscript. YQ and YO revised the manuscript. All authors contributed to the article and approved the submitted version.

## Conflict of Interest

The authors declare that the research was conducted in the absence of any commercial or financial relationships that could be construed as a potential conflict of interest.
